# Characteristics of general practice care: What do senior citizens value? A qualitative study

**DOI:** 10.1186/1471-2318-10-80

**Published:** 2010-11-02

**Authors:** P(Ine) GJ Berkelmans, Annette J Berendsen, Peter FM Verhaak, Klaas van der Meer

**Affiliations:** 1 Department of General Practice, University Medical Centre Groningen, University of Groningen, Antonius Deusinglaan 1, 9713 AV Groningen, The Netherlands

## Abstract

**Background:**

In view of the increasing number of senior citizens in our society who are likely to consult their GP with age-related health problems, it is important to identify and understand the preferences of this group in relation to the non-medical attributes of GP care. The aim of this study is to improve our understanding about preferences of this group of patients in relation to non-medical attributes of primary health care. This may help to develop strategies to improve the quality of care that senior citizens receive from their GP.

**Methods:**

Semi-structured interviews (N = 13) with senior citizens (65-91 years) in a judgement sample were recorded and transcribed verbatim. The analysis was conducted according to qualitative research methodology and the frame work method.

**Results:**

Continuity of care providers, i.e. GP and practice nurses, GPs' expertise, trust, free choice of GP and a kind open attitude were highly valued. Accessibility by phone did not meet the expectations of the interviewees. The interviewees had difficulties with the GP out-of-office hours services. Spontaneous home visits were appreciated by some, but rejected by others. They preferred to receive verbal information rather than collecting information from leaflets. Distance to the practice and continuity of caregiver seemed to conflict for respondents.

**Conclusions:**

Preferences change in the process of ageing and growing health problems. GPs and their co-workers should be also aware of the changing needs of the elderly regarding non-medical attributes of GP care. Meeting their needs regarding non-medical attributes of primary health care is important to improve the quality of care.

## Background

Senior citizens use medical care facilities more often than the young. This consumption of care increases significantly after the age of sixty [1]. Patients over 75 years are responsible for a large part of this increase [2].

In recent years, national and international research has been conducted on the non-medical service and product characteristics (attributes) of general practice (GP) care, and addressed how patients prioritize these [3-5]. Some pertinent research has been conducted among the general population to identify associations between GP care preferences and specific patient characteristics, including age. Priorities observed in the general population are e.g., quick service response to emergencies, adequate time for consultation, confidentiality of information about patients and informing patients adequately about their illness, offering preventive services, encouraging patients to feel free to talk about their problems and GPs attending refresher courses regularly[3]. Earlier research revealed that few studies have been conducted on the preferences of senior citizens [6-10].

These preferences may be dependent on age and deteriorating health status [6]. Compared to the young individuals, senior citizens are generally less healthy, suffer more frequently from multi-morbidity and are more dependent on other people [1]. Consequently, the preferences of senior citizens may differ from those of the general population [7-11].

Preferences are defined as ideas that individuals have about what they feel ought to be done (normative expectations) [12]. In order to be able to meet these expectations of older health care consumers, it is necessary for GPs to understand the way in which these expectations, preferences and priorities differ from those of the general population.

In view of the increasing number of senior citizens in our society who are likely to consult their GP with age-related health problems, it is important to identify and understand the preferences of this group in relation to the non-medical attributes of GP care, if we are to develop strategies to improve the quality of GP care for this group of patients.

In this study the following research question was investigated: *Which non-medical service and product attributes do senior citizens value in regard to GP care?*

## Methods

We opted for a qualitative explorative investigation, because little is known about the preferences that senior citizens value, we used semi-structured interviews to gain as many personal insights as possible about citizens with regard to the non-medical characteristics of GP care. To investigate the product and service attributes that senior the following items:

Continuity of care giver; distance to the practice; accessibility; expertise and trust; attitude; information; pro-active initiatives; free choice of selecting a caregiver; waiting times.

The topics are based on the results of earlier research [3-11]. Ethical approval was not required. In the Netherlands the "Medical Research Involving Human Subjects Act" (Wet Medisch-Wetenschappelijk Onderzoek met Mensen (WMO)) protects the interests of individuals that participate in medical research. Only medical research involving individuals subjected to the rules of conduct or acts, is liable for testing based on this law. Because our research is not within the scope of the WMO, no ethical approval was required.

### Study population

We chose to select a sample of individuals with diverge opinions for this investigation (judgement sample). The aim of this kind of sampling is to include individuals with a large variation in characteristics. A judgement sample is not representative of a particular population, but the individuals in the sample are involved in the circumstances that are being investigated [13]. We included patients with the assistance of several GPs from different practices whom we asked to select patients with different characteristics, e.g. gender, age, health, single/widowed/married and education level (see Table 1). The patients were selected from 4 GP practices (6 GPs) across the Netherlands: Limburg (southern and rural), Amsterdam (western and urban), Groningen (northern and urban) and Roden (northern and rural). All GP practices participate in a national research network related to various University Departments of General Practice. Patients in the same GP practice consulted different doctors. The actual interviews and analyses were conducted almost simultaneously, so that the researchers were able to control for topic saturation. We stopped recruiting when saturation was reached during the process of data analysis.

**Table 1:** Characteristics of the research population

	Age	Sex	Marital status	Education	Perceived health	Self-reliance GARS4
1	65	F	Divorced	Higher professional education	Good	18

2	74	M	Married	Primary school	Not so good	48

3	76	F	Married	Junior general secondary education	Quite good	18

4	77	F	Married	Junior general secondary education	Good	18

5	77	F	Widow	Primary school	Good	21

6	81	M	Widower	Higher professional education	Good	25

7	82	M	Married	Higher professional education	Very good	18

8	83	F	Married	Primary school	Not so good	45

9	83	M	Single	Higher professional education	Good	19

10	87	F	Widow	Senior secondary vocational education	Moderate	41

11	89	F	Married	Primary school	Moderate	38

12	90	M	Widower	Pre-university secondary education	Reasonable	37

13	91	M	Widower	Pre-university secondary education	Reasonable	21

The sample comprised 13 patients, 6 men and 7 women whose ages ranged from 65-91 years (mean age 81.2); 6 patients were married and living with their partner, 5 patients were widowed, 1 patient was divorced and 1 patient had never lived with a partner. The education level ranged from primary school to college/university. The variety in mobility was measured by The Groningen Activity Restriction Scale (GARS) and perceived health status. The GARS is a non-disease-specific instrument used to measure disability in activities of daily living (ADL) and instrumental activities of daily living (IADL)[14]. The score of the GARS version 4 varies from 18 (no restriction) to 48 (full assistance needed for all the activities). Perceived health status was scored on a five point scale varying from 'very good', 'good 'reasonable', 'moderate' to 'not so good'.

### Data collection

The GPs personally invited the patients to participate; all patients agreed; none of them declined. Subsequently an appointment for an interview was made with the patients by an independent interviewer. Three professional interviewers, all familiar with exploratory interviews, visited the patients at home. Before the interview began, the goal of the research project was explained to the interviewees and anonymity was guaranteed. The list of topics served as a check list for discussion, not necessarily to be followed in that order. The interviewer was allowed to follow the associations and order the participants chose, although all topics had to be addressed by the end of the interview.

### Analysis

Each interview was recorded and transcribed verbatim. Independently from each other, two researchers AJB (GP and senior researcher) and PGJB (researcher) conducted an analysis of the transcriptions and assigned labels to the most important statements. Disagreements between the two were resolved through discussion with each other after they had completed their independent rating.

During the next step, the researchers discussed any possible discrepancies until consensus was reached. The analysis was conducted according to the rules of the frame work method [15,16]. The five most important steps in this method are familiarization, identifying a thematic framework, indexing, charting and mapping/interpretation. The interviews and analysis were part of an iterative process in which the study team agreed on a preliminary coding frame by using initial interviews and broadly thematic content analytic approach [15,16]. A preliminary classification of the product and service attributes that senior citizens value was made after 4 interviews. The classification was adjusted after 8 and 12 interviews. The interviews and the analyses were conducted simultaneously. For data processing, we used Kwalitan 5.0 [17].

## Results

The interviews took place between November 2008 and October 2009 in the patient's home. Most of the interviews lasted one and a half hour. Saturation occurred during the tenth interview.

### Continuity of care giver

All interviewees valued the personal continuity of the caregiver highly. They appreciated the doctor having knowledge of their medical records and personal circumstances, which meant they did not have to tell their personal history repeatedly.

*Indeed, Madam, because he has been our doctor for so many years. I don't have to say much to him, really. He knows me inside out *(Woman, 87 years old).

Consulting their own GP was especially important if patients felt vulnerable. The interviewees had difficulties with the out-of-office hours services when care is delegated to a practice nurse and to other GPs on call, and not by a familiar GP or practice nurse.

*I was very confused and mixed up at the time. Yes, and that was a pity, not to have your own family doctor at a time like that *(Woman, 65 years old).

As well as personal continuity of their GP, the interviewees also reported that they find it important for the receptionist and practice nurse to know them. Hearing a familiar voice on the phone or seeing a familiar face at the practice made them feel at ease.

*And my wife likes the old assistant best, because she knows her from way back *(Man, 82 years old).

One patient mentioned that he prefers a practice nurse or a GP during the out-of office hours whose voices are familiar from when he calls the GP cooperative:

*I would prefer my GP to have an assistant who is also familiar with the patients, and that either of them answers the phone when I call *(Man, 74 years old).

For most patients, delegation of tasks to the practice nurse was not considered a problem: they assumed that the GP would only delegate tasks if he or she knew that the practice nurse was fully capable of performing the tasks. Some interviewees even considered delegation of tasks necessary to relieve the GP. Trust in the GP is an important factor in accepting that tasks are delegated.

*The assistants also measure blood pressure and that sort of thing. Not at all, it makes no difference to me. You see, he (the GP) shouldn't be burdened with doing everything himself *(Man, 74 years old).

Delegation of tasks was not acceptable for some of the interviewees. They only wanted to give information to the GP and emphatically asked for his advice.

*Because, nowadays, the receptionist, who is also the assistant, tries to solve it herself and I feel uncomfortable with that. "Yes, Madam, what are you calling for? Yes, Madam, what are you using?" I always say: Miss, I would like to speak to the doctor, please *(Woman, 87 years old).

The interviewees understood that, nowadays, doctors cannot be on duty 24 × 7, even though this was common practice in the past. The interviewees realize that quality of care is jeopardized if the doctor has not enough time to rest. They also stated that when they needed medical help or had an urgent question, they felt safe and liked the idea of always being able to speak to their own GP or, if he or she is absent, to a familiar colleague in the practice. This latter was acceptable to the interviewees as long as information about them was transferred adequately. Therefore they appreciated the observed increase in number of group practices.

*Now if you really need a doctor, then there will always be one on duty. That is the advantage of this group practice *(Man, 74 years old).

*I think it has a clear benefit to GPs to operate in a team of physicians in one place, they can inform each other, take over from each other, and in such a way that the patient's trust is never betrayed, whoever it may concern *(Man, 90 years old).

### Distance to the practice

Most interviewees valued having GP care in the neighbourhood, but continuity of caregiver seemed to interact with the willingness of a patient to pay a visit to the practice that is not within their vicinity. Sometimes the interviewee accepted the greater effort which was required to attend the practice, like taking a taxi or calling a family member to accompany him or her.

*I think... I sometimes ruminate: what should I do? Shall I consult this local doctor? And then I think: but he knows nothing about me. I am doubtful about this *(Woman, 87 years old).

For interviewees who were still able to drive, the distance to the practice was less important. In that instance good parking facilities were an issue.

*But at first it was easy to find a parking space. Accessibility, that is a problem, yes, especially for me, walking with a cane back and forth *(Man, 83 years old).

When his or her physical state impeded the patient from attending the practice, then the doctor was asked to pay home visits. Interviewees appreciated this service very much and almost all of them indicated that home visits should only be requested or offered when it is really necessary. They thought the doctor's time is precious and that this precious time should be reserved for patients in need. They also stated that home visits are more expensive and that they did not want to raise the costs of health care.

*Home visits are not really necessary, because I can still go there. You only learn to appreciate them if you can't go out anymore, of course *(Man, 91 years old).

### Accessibility

The interviewees found it important that GP care is easy to access.

Regarding accessibility by phone, the interviewees said that, if they could modify GP care, they would extend the times that the practice could be reached by phone and reduce the waiting time before the telephone call is answered.

*But those waits, aye, that is a shame. Or that you have to wait such a long time sometimes before you get them on the phone, because - all the employees - one or two, I think - but all the employees are busy, it is no fun *(Man, 83 years old).

*They have to attend meetings every morning at half past ten... And then they have their lunch break... but then I say, there should be a person available to operate the telephone, during meetings too *(Woman, 65 years old).

With regard to the waiting times for an appointment, the interviewees stated that it is important to consult the doctor on the same or the next day. Usually, they could make an appointment on the same or the next day and they were satisfied.

*....so, you can usually visit the doctor the following day or, if it's urgent, on the same day ... *(Man, 81 years old).

When they called the GP out-of-office hours service - in most cases they were in real need and very anxious at the time of the call - a short waiting time between the call and the consultation of a doctor became far more important. They indicated that when they call it is very important that they don't have to wait too long before their call is answered, and that the waiting time to speak to or see a doctor should be short too. It was clear the respondents lacked insight into the organizational and logistical processes of the out-of-office hours service.

*They should provide an adequate doctor's service during weekends. So that you don't have to wait too long or be kept up in the air ... or that communication with the doctor is not quick enough *(Man, 81 years old).

*That was horrible. They just didn't want to come when my husband... When you are so ill, and then you think, I am going to die *(Woman, 83 years old).

### Expertise and trust

The interviewees assumed that the GP is knowledgeable, but by this they meant different things i.e. knowledge of illnesses, knowledge of the patient (body and mind), making the right diagnosis, "seeing things you don't see yourself" and initiating quick referrals to medical specialists. Some interviewees said that they expected their GP to take postgraduate courses on a regular basis. Trust in the GP was very important for the interviewees; the assumption that the GP is knowledgeable is a basic condition for trust.

*I expect a doctor to take treatment seriously. That is very important to me. I also expect him to keep his level of knowledge up to par with his skills. By which I mean, that he takes refresher courses regularly *(Man, 81 years old).

*He is a bit older, so he is experienced and knowledgeable. And I have confidence in him to make the correct diagnosis *(Man, 74 years old).

### Attitude

All the interviewees reported that they highly valued a kind, open attitude. They wanted to feel free to discuss everything with their GP from health and relationship problems to end of life issues. The interviewees wanted to be treated with respect, they valued a GP who listens carefully and who gives them enough time and personal attention. They reiterated appreciating a GP who does not act in a superior manner. The sex and age of the doctor was not really an issue: some preferred a younger doctor; others suggested that it is nice to have a doctor with some life experience.

*An open mind and an atmosphere in which anything can be discussed and in which, for example, any different ideas that patients may have are taken seriously. Not one in which the doctor says in a superior manner: Well, we've got the picture now. No, it's important to be accessible and open minded towards each other *(Man, 90 years old).

*We also discussed euthanasia when I first saw her. I said: Doctor, there is one thing I want to know: Can I get help when my suffering becomes unbearable? She said: "I will always make sure that you do not suffer." Well, that says it all. *(Woman, 77 years old).

Allowing enough time for the consultation was an important aspect of attitude for the interviewees.

*I think it is important that the GP listens... takes time *(Man, 81 years old).

*I always ask for a double consultation. I do this deliberately and he has agreed. Otherwise, it is such a rush. For example, if he has to physically examine something... that is not done as quickly at our age. By the time you've finished fiddling about; it all takes more time *(Woman, 87 years old).

### Information

There are several ways of informing patients about their disease, medical treatment, medication and examinations. One interviewee said that she did not need any information at all, but all other interviewees liked to be informed. One of the interviewees said that he got all the information he needed from the internet.

In most practices, leaflets and brochures about diseases are available in the waiting room. The interviewees said they did not use these leaflets. They did not read the leaflets about prescribed medication either but preferred to receive information from their doctor verbally as long as they were able to remember the information afterwards. If their memories were declining they liked to receive written information from their own doctor as well.

*That he talks to me, yes, that he explains things. Let me tell you... in a folder you can have words to understand, difficult words *(Woman, 77 years old).

### Pro-active initiatives

Sometimes the GP just drops by. Interviewees who are very old or have very poor health mentioned this service. They appreciated it very much and considered the spontaneous visits as an expression of the GP's personal interest.

*The GP comes to visit me every month. He can easily stay for half an hour. Yes, that is quite ... that does me good *(Woman, 83 years old).

On the other hand, the interviewees who were in reasonably good health and had not experienced any spontaneous home visits were not unanimously enthusiastic. They found visits at home unnecessary if they were able to get to the practice and only wanted contact with the doctor on their own request. They also said that unnecessary visits push up the costs and are, therefore, unwanted.

*Oh no, that is not possible anymore these days. Well yes, if you are seriously ill... then *(Man, 91 years old).

### Waiting time in the waiting room

Most interviewees did not mind waiting fifteen minutes. If the waiting time was longer, then dissatisfaction grew. Comfort during the waiting time was important. The interviewees listed nice waiting-room design, bathroom facilities, space, reading matter and comfortable chairs as items that add to the comfort of the waiting room.

*If I have been sitting waiting for half an hour and have finished reading the magazines .... Yes, half an hour really is too long *(Woman, 65 years old).

*It is important to me, not having to wait too long, but oh well, what can you do about it? But I don't cope with it well. I know that... My blood pressure goes up so I have myself to blame for it *(Man, 91 years old).

### Free choice of GP

The interviewees valued highly the free choice in selecting a GP. They said that when they have no confidence in their GP they want to be able to switch to another one easily and without restriction. Some interviewees were prepared to pay extra to secure that right.

*It is worth something to have an agreeable GP. If you don't like your doctor... then the moment will come when you think, I wouldn't mind having to pay a little extra *(Man, 91 years old).

*In my opinion, you should decide yourself which GP to have... Because anybody... not everybody will appeal to you, so to say. And I have a good GP, I am very pleased to have this GP *(Woman, 77 years old).

## Discussion

### Continuity of caregiver

One of the conclusions of this study is that senior citizens find continuity of care giver very important. A familiar face is 'second best'. In the Netherlands the number of single-handed practices is decreasing rapidly. Most GPs work together with one or more colleagues in one practice; patients are registered with a particular GP and not with a practice. It is only when the GP with whom the patient is registered is not available, that a colleague in the practice fills in. The main reason the respondents value this system is because they feel safe and GP care is guaranteed. Usually patients are familiar with the names and faces of the colleagues in the practice. A familiar voice, face or name when they contact the practice appears to be very important for the interviewees and is used in combination with terms like 'trust', 'safe', 'personal' and 'nice'.

During out-of-office hours, practice nurses and GPs work on shifts and patients meet unfamiliar care givers. The respondents find that difficult: at the very time they are really worried their need for a familiar face or voice is not met.

### Distance to the practice

With aging it becomes more difficult to bridge the distance to the practice and a GP in the neighbourhood becomes more important than personal continuity. If necessary, GPs visit the patient at home. In that case, distance should not be a problem. The respondents argue that, as long as they are able, they would rather visit the practice themselves than to be visited by the doctor at home. This can be explained by the need for autonomy.

### Accessibility

The interviewees are satisfied with waiting times they have to endure before an appointment can be made; for this issue their expectations are met. Regarding continuity of care giver versus access to care, it is known from recent research that senior citizens find obtaining same-day health care less important than obtaining an appointment with their own doctor [18]. Research shows that, compared to the young, senior citizens have a greater preference for continuity of care[7,18-23]. The results of this study confirm this preference. Decisions have to be made regarding the trade-off between continuity of care giver and access to care: the need for continuity of care giver and access to care can (potentially) conflict in a practice which has a large number of doctors and a large number of tasks delegated to other employees such as nurse practitioners.

Accessibility by phone is very important to the interviewees and below their expectations. They find waiting times too long before a call is answered and the contact opportunities too limited during the day that the practice could be reached by telephone. If they had the chance, the interviewees would change this. Recent research in the Netherlands showed that in the top ten of the most important aspects for improvement regarding aspects of GP care 'getting some one on the phone within one minute' scored the highest for young patients as well as for the elderly [24].

### Attitude

All the interviewees find that the GP needs to have good communication skills to be able to discuss, the sometimes difficult, subjects. Adequate time for the consultation and respect for the patient are almost conditional for patients to feel free to speak openly. The importance of freedom to select their own GP is in line with this: if patients are not satisfied with the GP's attitude and feel restricted in speaking openly, they want to switch.

The interviewees wished to discuss their treatment with the GP and make their own decisions. Younger and more highly educated interviewees seem to exhibit a more pro-active attitude towards the doctor.

### Information

Conspicuously the interviewees showed a rather passive attitude towards obtaining information: they preferred to receive verbal information over collecting information contained within leaflets.

Elderly people appreciated a relationship that is focussed on the individual and not on the disease [19]. It is likely that because trust and support are considered very important in such a relationship, information from the person they trust, like the GP, is valued most.

In our sample, the interviewees were satisfied with the waiting times for an appointment. This can be explained by the results of recent studies that illustrate that, compared to the young, elderly people have lower expectations regarding GP care in general [10,19,23]. Moreover, use of same-day care decreases with age,[18] and that makes it easier to make an appointment at a more convenient time.

Based on the literature, we assumed senior citizens to value a higher level of involvement from the doctor in decision-making. [7,10,25,26]. It is conceivable that, compared to the patients examined in earlier cohorts, elderly patients in recent cohorts, who are more highly educated, have preferences that are more similar to the preferences of the young and value shared decision-making. It could also be related to the nature of the sample: a judgement sample.

### Pro-active initiatives

With ageing it becomes more difficult to bridge the distance between residence and practice and a GP in the neighbourhood becomes more important than continuity of care provided by the doctor of choice. If necessary, GPs visit the patient at home. In that case, distance should not be a problem. The respondents argue that, as long as they are able, they would rather visit the practice than welcome the doctor at their home. This can be explained by the need for autonomy and free choice of GP.

### Waiting time in the waiting room

Based on the literature, we also assumed senior citizens to have lower expectations regarding waiting times in the waiting room in comparison with young individuals. However, in the interviews, this was not confirmed. As for shared decision-making, our results for the waiting time could be due to the nature of the sample and cohort.

### Expertise and trust

From the interviews conducted, we deduce that the respondents were very reluctant to use the GP out-of-office hours services. They preferred to have no contact with an unknown caregiver. In a synthesis of the qualitative literature on patients' perspectives Ridd et al concluded that the (in depth) relationship with a GP is made up of primary values like knowledge, trust, loyalty and regard [27]. Trust in the care giver is an essential feature of the therapeutic relationship and related to an increased level of patient satisfaction [28,29]. It is possible that, in cases where the patient consults a GP on duty during out-of-office hours, trust is not established and patients are less likely to be satisfied.

Most interviewees lacked insight into the organizational structure of the GP out-of-office hours service. They do not know, for example, that the GP who is on duty has access to (a part of) the medical records. This could be another reason that they have less confidence in the unfamiliar GP on duty.

All interviewees valued a positive and sustainable relationship with their caregiver. This is not limited to the GP: continuity of the practice nurse was also important. Nowadays, because of task delegation and differentiation, the number of employees in a GP practice has grown. It is a challenge for GPs to strive for personal continuity of care to meet the needs of the elderly patient. Compared to the young, senior citizens have lower expectations regarding same day care and are more flexible. This is an opportunity to meet their stronger preference for personal continuity of care.

Waiting time before the telephone is answered is too long for senior citizens. To meet their (and other patients) needs at that point is not easy and probably entails an increase in the number of practice nurses.

GPs and their employees should be aware of senior citizens' need for autonomy and facilitate older people visits to the practice as long as possible. Simple facilities such as good parking places, 'walker friendly' access and comfortable waiting rooms would make that easier to materialize.

Most interviewees have had bad experiences with out-of-office hours. GPs on duty should, therefore, have access to a complete set of patient records. More information given to senior citizens about the logistical processes may lead to a greater understanding and acceptance of the provided out-of-office hours services.

#### Strengths and weaknesses

We selected patients with the help of the GPs. It is possible that the GPs selected patients with whom they have a good relationship, or patients with an assumed positive attitude towards the GP. This could have led to bias of results.

Some interviewees were healthy and did not consult their GP frequently. The interviews were taken from a customer perspective and were unrelated to illness. This is possibly the reason that half of the interviewees had very few contacts per year with their GP. We think that this sample - with a mix of healthy and unhealthy senior citizens - serves the preferences of healthy senior citizens more than a sample of unhealthy people would.

Since this is a qualitative study, the results should be regarded as exploratory.

The number of interviewees was limited: saturation was reached already after a small number of interviews. This could be due to the research population which was comprised solely of elderly patients. One reason could be that elderly people are more formed in their contacts with their GP and are less able to look across the boundary of the current 'model' [10]. Another explanation could be that senior citizens have lower expectations regarding health care [10,19,23]. They are satisfied with most aspects of the current GP health care services, and only raised the few attributes that are most important or that could be improved.

## Conclusions

The method we used proved to be useful. Regarding the role of the GP, waiting time in the waiting room and preference for the way information was given, our results differed from what we assumed based on the literature.

More knowledge of the weighted preferences of senior citizens is important if we are to meet their expectations of GP care. It is especially important to study the trade-off between the characteristics that are in potential conflict, such as access, the distance to the practice and continuity of care giver. A discrete choice experiment should be performed to elicit the preferences of senior citizens.

## Competing interests

The authors declare that they have no competing interests.

## Authors' contributions

PGJB, KM and AJB participated in planning and designing the study. All authors drafted the manuscript and approved the final version. AJB and PGJB performed the analyses; PGJB wrote the manuscript.

All authors read and approved the final text.

## Pre-publication history

The pre-publication history for this paper can be accessed here:

http://www.biomedcentral.com/1471-2318/10/80/prepub
